# Improvement of Three-Party Semi-Quantum Protocol for Deterministic Secure Quantum Dialogue Based on GHZ States

**DOI:** 10.3390/e27101002

**Published:** 2025-09-26

**Authors:** Ling Zhang, Xun Liu, Xiang-Jun Xin, Chao-Yang Li, Li Gong

**Affiliations:** College of Software Engineering, Zhengzhou University of Light Industry, Zhengzhou 450002, China; ll790217@163.com (L.Z.);

**Keywords:** semi-quantum dialogue, GHZ state, dishonest participant attack

## Abstract

Through the analysis of “Three-party semi-quantum protocol for deterministic secure quantum dialogue based on GHZ states”, we demonstrate that the protocol is vulnerable to attacks from dishonest participants. Specifically, the fully quantum-capable participant may behave dishonestly, leading the two semi-quantum participants to receive incorrect secret information, with the dishonest behavior remaining undetected. Accordingly, we propose an improved protocol that demonstrates robustness against various internal and external attacks, including dishonest participant attacks, and we further prove that it does not suffer from information leakage. Moreover, compared to the original protocol, the improved version achieves a significant enhancement in quantum communication efficiency.

## 1. Introduction

The quantum dialogue protocol is a secure communication protocol based on the principles of quantum communication. In 2004, Nguyen et al. [1] first introduced the concept of Quantum Dialogue (QD) protocols. By utilizing Bell states as the quantum channel, their protocol enabled two communicating parties to exchange secret messages simultaneously and securely, leveraging the entanglement property of Bell states. Shortly thereafter, in 2005, Man et al. [2] identified a critical vulnerability in Nguyen’s protocol [1], demonstrating that it could not resist intercept–resend attacks, and proposed an improved version to enhance its security. As research progressed, a variety of QD protocols were developed using different types of quantum states as communication channels, such as GHZ states [3,4] and single photons [5,6], thereby enriching the diversity and applicability of protocol designs. However, in 2008, Gao et al. [7] revealed that several existing protocols [2,3,5] suffer from information leakage, where an adversary can infer partial secret information based on the classical messages disclosed during the communication process. This finding brought significant attention to the problem of information leakage, which has since become a key consideration in the design of secure QD protocols [8,9,10].

Beyond conventional two-party communication, efforts were made to broaden the scope of QD protocols. In 2007, Xia et al. [11] proposed the Controlled Quantum Dialogue (CQD) protocol, introducing a third-party controller who supervises the dialogue between two legitimate communicators without directly participating in the transmission of secret information. Since then, CQD has become an important research branch in the field of QD, leading to a series of protocols with improved efficiency, security, and practical feasibility [12,13,14]. However, it is worth noting that although a third-party controller is introduced in some protocols, such as CQD, the aforementioned schemes remain fundamentally two-party in nature, as the controller does not engage in the exchange of secret messages. As a result, these protocols are not applicable to scenarios where all three participants wish to exchange secret information with one another. Fortunately, as research has progressed, quantum dialogue protocols have expanded beyond the limitation of only two participants. Many quantum dialogue protocols that allow for multi-party participation have been successfully designed. In these multi-party quantum dialogue protocols, all participants can share their information and obtain the secret information of the other participants. In 2016, Yu et al. [15] proposed a three-party quantum dialogue protocol based on continuous-variable GHZ states, which can be further extended to accommodate a larger number of communicating participants. In 2017, Cao et al. [16] introduced a multi-party quantum dialogue protocol based on multi-particle GHZ states. This protocol can be implemented using optical devices with high transmission efficiency, making it highly practical. Subsequently, in 2018, Gong et al. [17] proposed another multi-party quantum dialogue protocol based on continuous-variable GHZ states, which can also be more easily realized using controllable optical devices and has the potential to significantly enhance the channel capacity.

All of the above quantum dialogue protocols always require both participants to possess quantum capabilities. However, not every participant can afford expensive quantum devices. In 2007, Boyer et al. [18] proposed the first semi-quantum secret protocol, in which only one of the two participants possesses quantum capabilities, while the other one requires only classical abilities. Specifically, we distinguish between fully quantum participants, who can perform all quantum operations (including preparation, transmission, storage, and measurement in arbitrary bases), and semi-quantum participants, whose capabilities are restricted to simple operations such as preparing or measuring qubits in the computational basis, reflecting qubits without disturbance, and reordering them. This novel concept has since been applied to various quantum cryptography tasks, such as quantum key distribution (QKD) [19,20], quantum secure direct communication (QSDC) [21,22], and quantum secret sharing (QSS) [23,24]. In 2017, Shukla et al. [25] proposed the first semi-quantum dialogue protocol. Since then, a growing number of semi-quantum dialogue protocols have been developed to effectively reduce the reliance on quantum resources. Recent advancements include standard semi-quantum dialogue protocols [10,26,27] and multi-party semi-quantum dialogue protocols [28,29]. In the context of multi-party semi-quantum dialogue, Xu et al. [28] proposed the first three-party protocol based on cluster states in 2020, wherein two participants with only semi-quantum capabilities can successfully complete the dialogue, and the protocol exhibits relatively high efficiency.

Recently, a three-party semi-quantum dialogue quantum based on GHZ states was proposed by Zhou et al. [29] (Hereafter, we call this three-party SQD protocol the ZZL-SQD). This protocol allows each participant to share their secret and obtain the secrets of the other two participants, with one participant having full quantum capabilities and the other two participants having only semi-quantum abilities. Moreover, Zhou et al. have verified that the protocol can effectively resist various attacks, including intercept–measure attack, flip attack, man-in-the-middle attack, and entangle–measure attack; at the same time, it also demonstrates a high qubit efficiency. However, we will demonstrate that when the ZZL-SQD protocol is attacked by a dishonest participant, the two semi-quantum participants in the protocol will receive the wrong secret information about each other and will not detect the attack. Furthermore, we propose effective improvements to the ZZL-SQD protocol to defend against the aforementioned insider attack, while also enhancing its qubit efficiency. The main contributions of this work are summarized as follows:

(1) We identify a critical security flaw in the original ZZL-SQD protocol—specifically, its inability to defend against attacks launched by dishonest participants—and provide a detailed analysis accompanied by concrete examples to illustrate this vulnerability.
(2) To address potential attacks from dishonest participants, we propose an improved version of the ZZL-SQD protocol and conduct a comprehensive security analysis, demonstrating that the revised scheme is robust against various external and internal attacks and effectively prevents information leakage.
(3) We further evaluate the quantum communication efficiency of the proposed protocol. The results demonstrate a notable improvement over the original ZZL-SQD protocol, thereby enhancing its overall practicality and applicability.

The rest of this paper is arranged as follows: Section 2 gives a brief description of the ZZL-SQD protocol. Section 3 introduces the security analysis of ZZL-SQD protocol against dishonesty and presents an improvement. Section 4 analyzes its security and Section 5 provides the comparison. Finally, Section 6 makes a conclusion.

## 2. Description of the ZZL-SQD Protocol

Firstly, we briefly review the ZZL-SQD protocol. Suppose there are three participants: Alice, Bob, and Charlie. Each of them aims to share their information and obtain the secret information of the other participants. Alice, as the quantum participant, possesses complete quantum resources, while Bob and Charlie are semi-quantum participants with limited capabilities. The protocol consists of the following steps.

(1) $${|\Psi_{q_{1},q_{2},q_{3}}\rangle }_{ABC}=\frac{1}{\sqrt{2}}{\left(|q_{1},q_{2},q_{3}\rangle +{\left(-1\right)}^{\Delta }|\bar{q_{1},q_{2},q_{3}}\rangle \right)}_{ABC}$$

Initialization: Alice, Bob, and Charlie each have *n* bits of secret information, denoted as $m_{A}$, $m_{B}$ and $m_{C}$, respectively.

**Step 1**: Alice randomly prepares *n* GHZ states according to Equation (1) and obtains a sequence of GHZ states, denoted as S, where $q_{1}=0$, $q_{2},q_{3}\in \left\{0,1\right\}$ and $\Delta \in \left\{0,1\right\}$. She then partitions each GHZ state into three particles: $S_{A}$, $S_{B}$, and $S_{C}$. Next, Alice randomly prepares 4*n* decoy photons using measurement bases X or Z. She inserts 2*n* decoy photons into sequences $S_{B}$ and $S_{C}$, resulting in two new particle sequences, ${\bar{S}}_{B}$ and ${\bar{S}}_{C}$. Alice keeps $S_{A}$ and sends ${\bar{S}}_{B}$ and ${\bar{S}}_{C}$ to Bob and Charlie, respectively.

**Step 2**: After Bob and Charlie receive the sequence, respectively, Alice announces the positions of all the decoy photons in ${\bar{S}}_{B}$ and ${\bar{S}}_{C}$. Bob and Charlie use quantum delay line to temporarily store sequences and then select the first *n* decoy photons from the 2*n* ones, which they received to conduct the first eavesdropping check. They randomly perform one of the following two operations on the decoy photons:

(1) M: Measure the decoy photon using the Z-basis and prepare a photon in the same state as the measurement result, then send it back to Alice.
(2) R: Directly reflect the photon to Alice without making any disturbance.

**Step 3**: Once Alice confirms that she has received all the returned decoy photons, Bob and Charlie announce the positions of the corresponding *n* decoy photons, the operations performed, and the measurement results, respectively. Alice then performs the first eavesdropping check. For the photons on which the M operation was performed, Alice measures the received decoy photons using the Z-basis, compares the measurement results with those announced by Bob and Charlie, and calculates the error rate. For the photons on which the R operation was performed, Alice measures them using the basis she originally used when preparing the corresponding photons and calculates the error rate. If the overall error rate exceeds a predefined threshold, the protocol is terminated and the communication is aborted; otherwise, the protocol continues.

**Step 4**: Bob and Charlie each separate the remaining *n* decoy photons from ${\bar{S}}_{B}$ and ${\bar{S}}_{C}$, then obtain sequences $S_{B}$ and $S_{C}$, respectively. Then, Bob (Charlie) performs Z-basis measurements on the particles in $S_{B}$($S_{C}$) and records the measurement results. Next, Bob (Charlie) encrypts the information particle sequence according to their secret information $m_{B}$($m_{C}$) using the following encryption rule:

(1) If $m_{B}^{i}$($m_{C}^{i}$) is 1, Bob (Charlie) prepares a state in the Z-basis that is the opposite of the measurement result of the corresponding particle $S_{B}^{i}$($S_{C}^{i}$).
(2) If $m_{B}^{i}$($m_{C}^{i}$) is 0, Bob (Charlie) does nothing.

After Bob (Charlie) completes the encryption process, the sequence $S_{B}$($S_{C}$) is transformed into $S_{B}^{\prime }$($S_{C}^{\prime }$). For the remaining *n* decoy photons, Bob (Charlie) randomly performs one of the following two operations:

(1) MM: Measure the decoy photon using the Z-basis and prepare a photon in the same state as the measurement result.
(2) RR: Do nothing; Once all decoy photons have been processed, Bob (Charlie) reorders all the particles in the sequence $S_{B}^{\prime }$($S_{C}^{\prime }$) to obtain a new sequence $S_{B}^{\prime \prime }$($S_{C}^{\prime \prime }$). Bob and Charlie then send $S_{B}^{\prime \prime }$ and $S_{C}^{\prime \prime }$ to Alice, respectively.

**Step 5**: Similar to step 3, Alice performs the second eavesdropping detection on sequences $S_{B}^{\prime \prime }$ and $S_{C}^{\prime \prime }$, finally calculates the error rate and determines whether to terminate the protocol.

**Step 6**: After the second eavesdropping check is passed, Bob and Charlie announce the correct order of the information particles in the sequences $S_{B}^{\prime \prime }$ and $S_{C}^{\prime \prime }$ to Alice. This allows Alice to recover the sequences $S_{B}^{\prime }$ and $S_{C}^{\prime }$ after removing the decoy photons. Alice performs measurements on the particle sequences $S_{A}$, $S_{B}^{\prime }$, and $S_{C}^{\prime }$ in the Z-basis, obtaining the corresponding measurement result sequences $R_{A}$, $R_{B}^{\prime }$ and $R_{C}^{\prime }$. Ultimately, Alice can deduce Bob’s and Charlie’s secret information. Alice then announces the values of $R_{A}⊕R_{B}^{\prime }⊕R_{C}^{\prime }$ and $m_{A}⊕R_{A}$, and reveals the initial GHZ states based on the positions of each particle in the sequences $S_{B}$ and $S_{C}$. Consequently, Bob or Charlie can infer the secret information of the other two participants based on the information published by Alice.

## 3. Analysis and Improvement of ZZL-SQD Protocol

### 3.1. Security Analysis of ZZL-SQD Protocol Against the Dishonest Participant Attack

Zhou et al. proved that their protocol can resist various attacks, such as flip attacks, intercept–measure attacks, man-in-the-middle attacks, and entangle–measure attacks. The analysis showed that the protocol can both avoid information leakage problems and detect a variety of attacks employed by external adversaries like Eve. However, they did not consider the possibility of an internal dishonest participant attack, in which one participant in the protocol may have dishonest behavior to cause other participants to receive incorrect secret information, and such dishonest behavior would not be detected.

For the ZZL-SQD protocol, Alice could potentially become a dishonest participant, and she can keep Bob and Charlie from getting the correct secret information about each other by announcing false classic information.

Here, we provide an example. Suppose the initial GHZ state prepared by Alice is ${|\Psi_{000}\rangle }_{ABC}=\frac{1}{\sqrt{2}}{\left(|000\rangle +|111\rangle \right)}_{ABC}$, and the secret information of Alice, Bob, and Charlie is 0, 1, and 1, respectively. Bob and Charlie will measure the received information particles and then prepare states opposite to the measurement results, which are then inserted into the sequences $S_{B}^{\prime }$ and $S_{C}^{\prime }$. Assume the initial GHZ state ${|\Psi_{000}\rangle }_{ABC}$ collapses into the state ${|000\rangle }_{ABC}$ after Bob and Charlie’s measurements. After completing the second eavesdropping check, Alice performs Z-basis measurements on her particles, and the information particles encoded by Bob and Charlie, the resulting state will be ${|011\rangle }_{ABC}$. From this, Alice can infer that both Bob’s and Charlie’s secret information is 1 by performing an XOR operation, as shown in Equations (2) and (3).

(2) $$m_{B}^{i}=R_{B}^{i}⊕{R_{B}^{i}}^{\prime }=0⊕1=1$$

(3) $$m_{C}^{i}=R_{C}^{i}⊕{R_{C}^{i}}^{\prime }=0⊕1=1$$

Then Alice announces the initial GHZ state is ${|\Psi_{000}\rangle }_{ABC}$, as well as the values of $R_{A}^{i}⊕{R_{B}^{i}}^{\prime }⊕{R_{C}^{i}}^{\prime }$ and $m_{A}^{i}⊕R_{A}^{i}$, as shown in Equations (4) and (5).

(4) $$R_{A}^{i}⊕{R_{B}^{i}}^{\prime }⊕{R_{C}^{i}}^{\prime }=0⊕1⊕1=0$$

(5) $$m_{A}^{i}⊕R_{A}^{i}=0⊕0=0$$

For Bob, after performing Z-basis measurements on his information particles, he obtains the result $R_{B}^{i}=0$. Therefore, he can deduce that the entangled state ${|\Psi_{000}\rangle }_{ABC}$ collapsed to ${|000\rangle }_{ABC}$, and subsequently determine $R_{A}^{i}=0$ and $R_{C}^{i}=0$. Bob can then calculate Charlie’s secret information based on the value of $m_{A}^{i}⊕R_{A}^{i}$, $R_{A}^{i}$, and $R_{C}^{i}$, as shown in Equation (6).

(6) $$m_{C}^{i}={R_{C}^{i}}^{\prime }⊕R_{C}^{i}=\left(R_{A}^{i}⊕{R_{B}^{i}}^{\prime }⊕{R_{C}^{i}}^{\prime }\right)⊕R_{A}^{i}⊕{R_{B}^{i}}^{\prime }⊕R_{C}^{i}=0⊕0⊕1⊕0=1$$

Bob can also calculate Alice’s secret information based on the values of $m_{A}^{i}⊕R_{A}^{i}$ and $R_{A}^{i}$, as shown in Equation (7).

(7) $$m_{A}^{i}=\left(m_{A}^{i}⊕R_{A}^{i}\right)⊕R_{A}^{i}=0⊕0=0$$

Charlie obtains Bob’s and Alice’s secret information using a process similar to the one described above, so the details are not repeated here.

However, if Alice is dishonest and publishes 1 as the value of $R_{A}^{i}⊕{R_{B}^{i}}^{\prime }⊕{R_{C}^{i}}^{\prime }$, which is the opposite of the value of Equation (4), the corresponding Bob can deduce that Charlie’s secret value is 0, as shown in Equation (8).

(8) $$m_{C}^{i}={R_{C}^{i}}^{\prime }⊕R_{C}^{i}=\left(R_{A}^{i}⊕{R_{B}^{i}}^{\prime }⊕{R_{C}^{i}}^{\prime }\right)⊕R_{A}^{i}⊕{R_{B}^{i}}^{\prime }⊕R_{C}^{i}=1⊕0⊕1⊕0=0$$

Charlie deduces that the value of Bob’s secret is 0, as shown in Equation (9).

(9) $$m_{B}^{i}={R_{B}^{i}}^{\prime }⊕R_{B}^{i}=\left(R_{A}^{i}⊕{R_{B}^{i}}^{\prime }⊕{R_{C}^{i}}^{\prime }\right)⊕R_{A}^{i}⊕{R_{C}^{i}}^{\prime }⊕R_{B}^{i}=1⊕0⊕1⊕0=0$$

Ultimately, Bob and Charlie get the wrong secret information about each other because of Alice’s operation. They didn’t know that Alice had published false information, because Alice had more responsibility and authority than Bob and Charlie, yet Bob and Charlie couldn’t be sure that Alice was acting honestly. Specifically, Bob and Charlie just perform different operations on the received particles and then return them to Alice, who is responsible for performing the two rounds of eavesdropping checks and announcing the classical information. After completing the second eavesdropping check, Alice can deduce Bob’s and Charlie’s secret information first, then publish the valid classic information and let Bob and Charlie calculate the secret information. However, to obtain the secret information about Alice and Charlie (Bob), Bob (Charlie) must rely entirely on the classical information announced by Alice, but he cannot verify the correctness of the classical information published by Alice and the secret information calculated by himself. This allows Alice to modify the public classical information, making it easy for Bob and Charlie to obtain the tampered secret information, and the attack can be successfully executed without being detected by Bob and Charlie.

### 3.2. Improvement of ZZL-SQD Protocol

**Figure 1:**
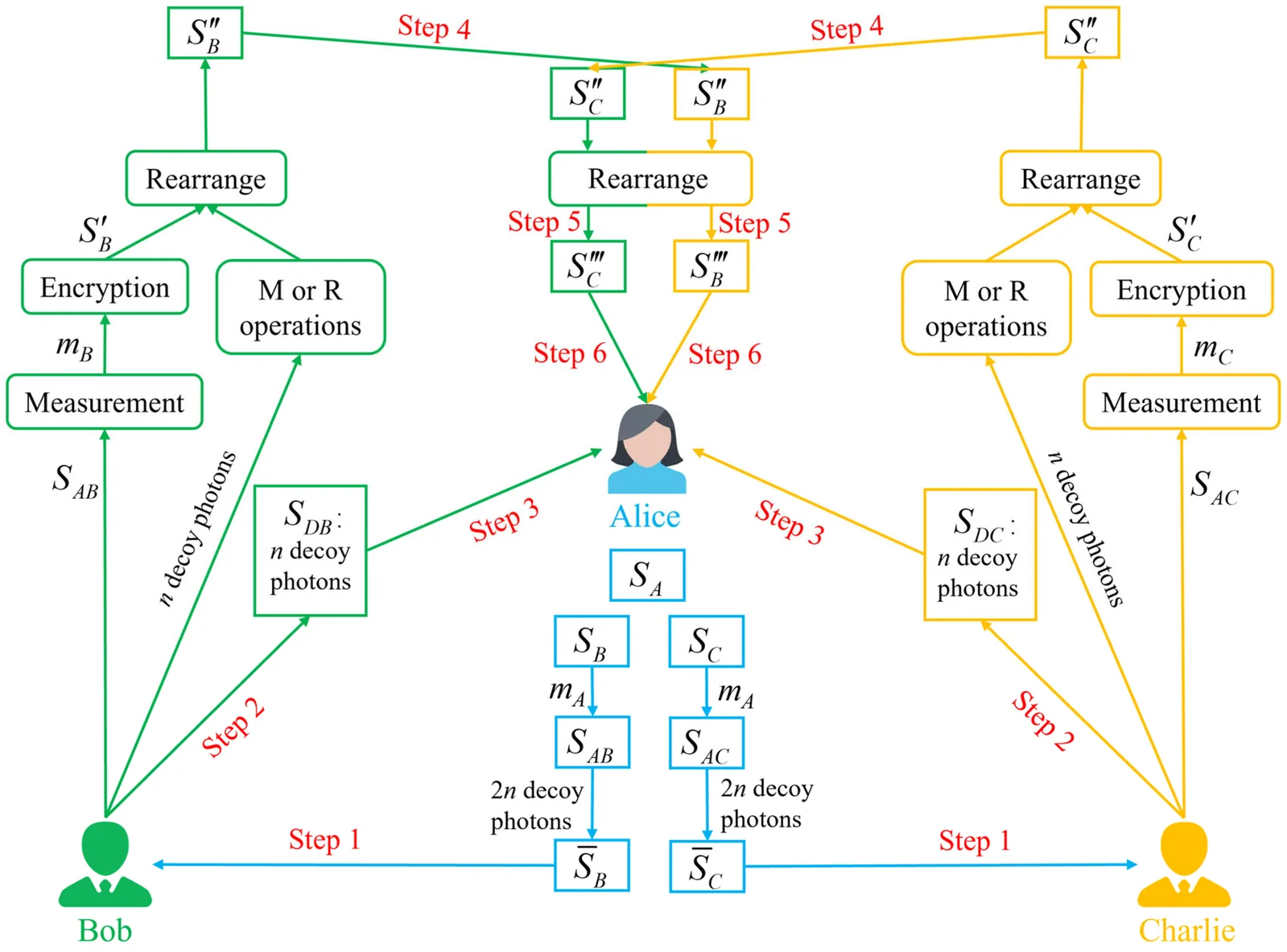
The schematic diagram of the improved ZZL-SQD protocol.

In the ZZL-SQD protocol, Alice is assumed to be a trusted participant. However, considering the crucial need to enhance security against the potential dishonesty of any participant in semi-quantum communication protocols [30,31], we propose an improved protocol that eliminates the requirement for a trusted Alice. The schematic diagram of the improved ZZL-SQD protocol is shown in Figure 1. Before the protocol begins, all participants use a one-way hash function $H:{\left\{0,1\right\}}^{*}\to {\left\{0,1\right\}}^{n}$. The content of the improved protocol is as follows:

**Step1**: Alice randomly prepares *n* GHZ states according to Equation (1) and obtains a sequence of GHZ states, denoted as S, where $q_{1}=0$, $q_{2},q_{3}\in \left\{0,1\right\}$ and $\Delta \in \left\{0,1\right\}$, and where the value of *n* is a public parameter known to all participants. She then partitions the particles according to their positions into three separate information particle sequences: $S_{A}$, $S_{B}$, and $S_{C}$, corresponding to the first, second, and third particles of each GHZ state, respectively. Next, Alice performs encryption operations on the particle sequences $S_{B}$ and $S_{C}$ based on the *n*-long binary secret information $m_{A}$, resulting in the new sequence $S_{AB}$ and $S_{AC}$, respectively. The encryption rule is defined as follows: if $m_{A}^{i}=0\left(i\in n\right)$, no operation is applied to the corresponding particles $S_{B}^{i}$ and $S_{C}^{i}$; if $m_{A}^{i}=1\left(i\in n\right)$, a Pauli-X operation ($\delta_{X}$) is applied to both particles $S_{B}^{i}$ and $S_{C}^{i}$. The Pauli-X operation flips the state of a qubit, e.g., $\delta_{X}|0\rangle =|1\rangle ,\delta_{X}|1\rangle =|0\rangle$. She then randomly generates 4*n* decoy photons in either the X- or Z-basis, and inserts 2*n* of them into the sequences $S_{AB}$ and $S_{AC}$, respectively, resulting in the modified sequences ${\bar{S}}_{B}$ and ${\bar{S}}_{C}$. Finally, Alice keeps $S_{A}$ and sends ${\bar{S}}_{B}$ and ${\bar{S}}_{C}$ to Bob and Charlie, respectively.

**Step 2**: Upon Bob (Charlie) receiving each particle from the sequence ${\bar{S}}_{B}$(${\bar{S}}_{C}$), Alice announces whether the particle at the corresponding position is a decoy photon. For the first *n* decoy photons in ${\bar{S}}_{B}$(${\bar{S}}_{C}$), Bob and Charlie randomly choose to perform one of the following two operations:(1) M: Measure the decoy photon in the Z-basis and prepare a new photon in the corresponding measured state; (2) R: Perform no operation. By employing quantum delay lines, Bob (Charlie) then applies a reordering operation to these *n* decoy states to form the sequence $S_{DB}$($S_{DC}$). Finally, Bob and Charlie respectively send $S_{DB}$ and $S_{DC}$ to Alice.

**Step 3**: This step is identical to Step 3 of the original ZZL-SQD protocol.

**Step 4**: Based on the information announced by Alice, Bob (Charlie) can distinguish the information particles from the remaining *n* decoy photons in the sequence ${\bar{S}}_{B}$(${\bar{S}}_{C}$). For the information particles, Bob (Charlie) performs Z-basis measurements on the information particles in $S_{AB}$($S_{AC}$) and records the measurement results. Next, Bob (Charlie) encrypts the information particle sequence according to their secret information $m_{B}$($m_{C}$) using the following encryption rule: (1): If $m_{B}^{i}$($m_{C}^{i}$) is 1, Bob (Charlie) prepares a state in the Z-basis that is the opposite of the measurement result of the corresponding particle $S_{B}^{i}$($S_{C}^{i}$). (2): If $m_{B}^{i}$($m_{C}^{i}$) is 0, Bob (Charlie) does nothing. After Bob (Charlie) completes the encryption process, the sequence $S_{AB}$($S_{AC}$) is transformed into $S_{B}^{\prime }$($S_{C}^{\prime }$). For the remaining n decoy photons, Bob and Charlie randomly perform either the R or M operation as described in Step 2. By employing quantum delay lines, Bob (Charlie) reorders all decoy photons and the particles in the sequence $S_{B}^{\prime }$($S_{C}^{\prime }$) to obtain sequence $S_{B}^{\prime \prime }$($S_{C}^{\prime \prime }$). Eventually, Bob and Charlie send $S_{B}^{\prime \prime }$ and $S_{C}^{\prime \prime }$ to each other.

**Step 5**: Upon Charlie receiving each particle from the sequence $S_{B}^{\prime \prime }$, Bob announces whether the particle at the corresponding position is an information particle. Charlie only performs Z-basis measurements on the information particles in $S_{B}^{\prime \prime }$ and records the corresponding results. Ultimately, Charlie rearranges all the information particles and decoy photons to obtain the new sequence $S_{B}^{\prime \prime \prime }$. Similarly, upon Bob receiving each particle from the sequence ${S^{\prime \prime }}_{C}$, Charlie announces whether the particle at the corresponding position is an information particle. Bob only performs Z-basis measurements on the information particles in $S_{C}^{\prime \prime }$ and records the corresponding results. Ultimately, Bob rearranges all the information particles and decoy photons to obtain the new sequence $S_{C}^{\prime \prime \prime }$. Bob and Charlie then send $S_{B}^{\prime \prime \prime }$ and $S_{C}^{\prime \prime \prime }$ to Alice, respectively.

**Step 6**: After confirming Alice has received $S_{B}^{\prime \prime \prime }$ and $S_{C}^{\prime \prime \prime }$, Bob and Charlie announce the positions, operations and measurement results of the corresponding *n* decoy photons in the sequences, respectively. Similar to step 3, Alice performs the same operation on these decoy photons as the first eavesdropping detection, then she calculates the error rate and determines whether to terminate the protocol.

**Step 7**: After the second eavesdropping check is passed, Bob and Charlie announce the correct order of the information particles in $S_{B}^{\prime \prime \prime }$ and $S_{C}^{\prime \prime \prime }$ to Alice. This allows Alice to recover the sequences $S_{B}^{\prime }$ and $S_{C}^{\prime }$ after removing the decoy photons. Then, Alice performs Z-basis measurements on the particles in sequences $S_{A}$, ${S^{\prime }}_{B}$, and $S_{C}^{\prime }$, obtaining the corresponding measurement result sequences $R_{A}$, $R_{B}^{\prime }$ and $R_{C}^{\prime }$. Alice announces the values of $H(R_{B}^{\prime })$ and $H(R_{C}^{\prime })$, which are verified by Bob and Charlie for correctness. If both are correct, Alice can deduce the correct secret information of Bob and Charlie. Alice then announces the values of $R_{A}$ and reveals the initial GHZ states based on the positions of each particle in the sequences $S_{B}$ and $S_{C}$. Consequently, Bob or Charlie can infer the secret information of the other two participants based on the information published by Alice.

## 4. Security Analysis

The quantum dialogue protocol enables communication between participants over a quantum channel. However, during this process, potential adversaries may launch various attacks to intercept the transmission of secret information. These adversaries are typically categorized into two types: external adversaries and internal adversaries (dishonest participants). Internal adversaries were first introduced by Gao et al. in 2007 [32]. Gao et al. emphasized that dishonest participants possess greater capabilities than external attackers, making them more likely to either steal secret information from other participants or manipulate the accuracy of the information received by others, all without detection. Moreover, in contrast to the original protocol, our improved version introduces an additional step wherein Bob and Charlie exchange particle sequences via a dedicated quantum channel. This modification, however, introduces potential vulnerabilities to previously negligible or irrelevant attacks—including, but not limited to, intercept–resend, measurement–resend, and Trojan horse attacks specifically targeting the Bob-Charlie link. In this section, we conduct a security analysis of the improved protocol, focusing on two critical aspects: external attack and dishonest participant attack. Additionally, an analysis of potential information leakage is also essential to ensure the protocol’s robustness.

### 4.1. External Attack

An external attacker (commonly denoted as Eve) is assumed to possess full quantum capabilities. By eavesdropping on the quantum channel, Eve may launch various types of attacks, including the intercept–prepare–resend attack, intercept–measure-resend attack, entangle–measurement attack, and Trojan horse attack, in an attempt to eavesdrop on or interfere with the communication process.

#### 4.1.1. Intercept–Prepare–Resend Attack

In an intercept–prepare–resend attack, the external eavesdropper Eve first prepares a set of fake single-particle states in either the X-basis or the Z-basis. She then performs eavesdropping by intercepting the quantum channel between any two participants. For each qubit in the quantum sequence transmitted from the sender to the receiver, Eve discards the original particle and replaces it with one of her own fake particles, which is then forwarded to the intended recipient. Since Eve does not perform any measurement on the intercepted particles, she avoids directly disturbing the entangled state’s collapse. However, in the proposed improved protocol, such an attack inevitably introduces detectable errors. The core reason lies in the protocol’s use of decoy photons randomly prepared in the four quantum states $\left\{|0\rangle ,|1\rangle ,|+\rangle ,|-\rangle \right\}$. Bob and Charlie randomly perform either R or M operation on these decoy photons, each with equal probability of 1/2. Taking Bob as an example: When Bob performs the R operation, Eve has a 1/4 probability of having prepared a state that is identical to the original decoy photon, which would not introduce any error. She also has a 1/4 chance of preparing the orthogonal state, which will certainly result in a detectable error when Alice performs her measurement. Additionally, with a 1/2 probability, Eve prepares a non-orthogonal state (e.g., the original decoy photon is $|+\rangle$, but the fake state is $|0\rangle$ or $|1\rangle$), which introduces an error with a probability of 1/2 upon Alice’s measurement. When Bob performs the M operation, similar statistical outcomes apply. Eve has a 1/4 chance of correctly guessing and preparing the same state as Bob’s resending, introducing no error. She also has a 1/4 probability of preparing the orthogonal state, which causes a definite error. In the remaining 1/2 of cases, Eve prepares a non-orthogonal state (e.g., Bob resends $|0\rangle$, but Eve replaces it with $|+\rangle$ or $|-\rangle$), which leads to an error with a probability of 1/2 during Alice’s measurement.

In summary, for the *n* decoy photon used in each eavesdropping detection process, the probability that Eve will be detected by launching an intercept–prepare–resend attack is $1-{\left(2\times \frac{1}{2}\times \left(\frac{1}{4}\times 1+\frac{1}{2}\times \frac{1}{2}\right)\right)}^{n}=1-{\left(\frac{1}{2}\right)}^{n}$.

#### 4.1.2. Intercept–Measure–Resend Attack

In an intercept–measure-resend attack, Eve performs eavesdropping on the quantum channel between any two participants. For each particle in the quantum sequence transmitted from the sender to the receiver, Eve intercepts the particle and measures it in either the Z-basis or the X-basis. She then prepares a new particle in the measured state and sends it to the intended recipient. However, under the proposed improved protocol, such an attack inevitably introduces detectable errors due to the presence of decoy photons. Specifically, decoy photons are randomly prepared in one of the four states $\left\{|0\rangle ,|1\rangle ,|+\rangle ,|-\rangle \right\}$, and Bob and Charlie randomly choose to perform either R or a M operation on each received photon, with equal probability. Taking Bob as an example: If Bob performs the R operation, Eve has a 1/2 chance of selecting the same basis as Alice’s preparation basis. In this case, the measured state remains consistent with the original, and no error is introduced. However, with the remaining 1/2 probability, Eve measures the particle in the opposite basis (e.g., the decoy photon is prepared in $|+\rangle$, but Eve uses the Z-basis). In such cases, there is a 1/2 chance that Alice’s final measurement will detect an inconsistency, thereby revealing Eve’s presence; If Bob performs the M operation, the decoy photon will eventually collapse into one of the computational basis states, $|0\rangle$ or $|1\rangle$, and Bob resends the result to Alice. If Eve uses the Z-basis to measure the photon, her action does not alter the outcome and no error is introduced. Conversely, if she uses the X-basis for measurement, there will be a 1/2 probability that the state she resends leads to an error when Alice performs her measurement.

In summary, for the *n* decoy photon used in each eavesdropping detection process, the probability that Eve will be detected by launching an intercept–measure-resend attack is $1-{\left(2\times \frac{1}{2}\times \left(\frac{1}{2}\times 1+\frac{1}{2}\times \frac{1}{2}\right)\right)}^{n}=1-{\left(\frac{3}{4}\right)}^{n}$.

#### 4.1.3. Entangle–Measurement Attack

In an entangle–measurement attack, the eavesdropper Eve does not directly measure or tamper with the particles transmitted through the quantum channel. Instead, she applies a unitary operation $U_{E}$ that entangles each transmitted particle with an auxiliary particle $|E\rangle$ of her own. This strategy enables Eve to delay her measurement and optimize it later based on the classical information publicly announced during the protocol, thereby increasing her chance of extracting useful secret information. However, in the proposed improved protocol, Eve is unaware of the exact positions of the decoy photons. To maximize the effectiveness of her attack, she must apply the entangling operation $U_{E}$ indiscriminately to all transmitted particles, including both decoy and information particles. Suppose the transmitted qubit is in the state $|0\rangle$ or $|1\rangle$; the unitary operation $U_{E}$ acts as follows:

(10) $$\begin{array}{c} U_{E}\left(|0\rangle |E\rangle \right)=\alpha_{0}|0\rangle |E_{00}\rangle +\beta_{0}|1\rangle |E_{01}\rangle \\ U_{E}\left(|1\rangle |E\rangle \right)=\alpha_{1}|0\rangle |E_{10}\rangle +\beta_{1}|1\rangle |E_{11}\rangle \end{array}$$

The parameters $\alpha_{0}$ and $\beta_{0}$, as well as $\alpha_{1}$ and $\beta_{1}$, satisfy ${\left|\alpha_{0}\right|}^{2}+{\left|\beta_{0}\right|}^{2}=1$ and ${\left|\alpha_{1}\right|}^{2}+{\left|\beta_{1}\right|}^{2}=1$, respectively. To avoid introducing errors during eavesdropping detection, Eve must ensure that the entangled quantum state remains unchanged, which requires $\beta_{0}=\alpha_{1}=0$. Thus, we obtain Equation (11):

(11) $$\begin{array}{c} U_{E}\left(|0\rangle |E\rangle \right)=\alpha_{0}|0\rangle |E_{00}\rangle \\ U_{E}\left(|1\rangle |E\rangle \right)=\beta_{1}|1\rangle |E_{11}\rangle \end{array}$$

When the transmitted particle is $|+\rangle$ or $|-\rangle$, it follows from Equation (11) that the unitary operation $U_{E}$ acts on these states as:

(12) $$\begin{array}{ll} U_{E}\left(|+\rangle |E\rangle \right) & =U_{E}\left(\frac{|0\rangle +|1\rangle }{\sqrt{2}}|E\rangle \right)=\frac{1}{\sqrt{2}}\left(\alpha_{0}|0\rangle |E_{00}\rangle +\beta_{1}|1\rangle |E_{11}\rangle \right)\\ & =\frac{1}{2}\left[\left(\alpha_{0}|E_{00}\rangle +\beta_{1}|E_{11}\rangle \right)|+\rangle +\left(\alpha_{0}|E_{00}\rangle -\beta_{1}|E_{11}\rangle \right)|-\rangle \right]\\ U_{E}\left(|1\rangle |E\rangle \right) & =U_{E}\left(\frac{|0\rangle -|1\rangle }{\sqrt{2}}|E\rangle \right)=\frac{1}{\sqrt{2}}\left(\alpha_{0}|0\rangle |E_{00}\rangle -\beta_{1}|1\rangle |E_{11}\rangle \right)\\ & =\frac{1}{2}\left[\left(\alpha_{0}|E_{00}\rangle -\beta_{1}|E_{11}\rangle \right)|+\rangle +\left(\alpha_{0}|E_{00}\rangle +\beta_{1}|E_{11}\rangle \right)|-\rangle \right] \end{array}$$

Clearly, if Eve intends to avoid introducing errors during the subsequent eavesdropping detection, her entangling operation must ensure that the quantum state of the transmitted particle remains unchanged. This requirement leads to the condition expressed in Equation (13).

(13) $$\alpha_{0}|E_{00}\rangle =\beta_{1}|E_{11}\rangle$$

Based on Equations (11)–(13), it can be concluded that if Eve’s attack is to remain undetectable, the measurement outcomes on her auxiliary particles must be statistically independent of the quantum states transmitted between the legitimate participants. This implies that Eve cannot extract any meaningful information about the secret messages from her entangled ancillary system. Therefore, the proposed improved SQD protocol is secure against entangle measurement attack.

#### 4.1.4. Trojan Horse Attack

On the quantum channel between Alice and Bob, and between Alice and Charlie, Eve eavesdrops by launching two common Trojan Horse attacks, invisible photon attacks [33], and delayed photon attacks [34]. Nevertheless, Alice, Bob, and Charlie can detect these attacks using filter and photon number splitter techniques before measuring or reflecting on the particles they receive.

### 4.2. Dishonest Participant Attack

In the original ZZL-SQD protocol, Alice, as a potentially dishonest participant, may attempt to interfere with the dialogue between Bob and Charlie by manipulating the quantum communication process. In contrast, under the improved protocol proposed in this study, if Alice intends to achieve the same objective, her dishonest behavior would have to occur in one of the following two scenarios: (1) launching an attack on the quantum channel during Step 4, when Bob and Charlie exchange their encrypted sequences; (2) publishing false classical information during Step 7 in an attempt to mislead the recovery of secret messages. The following provides a rigorous analysis of both scenarios, demonstrating that under the improved protocol, Alice’s dishonest intentions cannot be successfully realized.

For the first case, consider the transmission of the quantum sequence $S_{B}^{\prime \prime }$ from Bob to Charlie. If Alice intends to compromise Charlie’s recovery of Bob’s secret information, she must first extract the encrypted content from $S_{B}^{\prime \prime }$ and then alter the corresponding quantum states. However, in Step 4, the sequence $S_{B}^{\prime \prime }$ is generated by Bob through randomly rearranging a mixture of decoy photons and information particles. The exact positions of the information qubits are disclosed only after Charlie has acknowledged receipt of each individual particle. As analyzed in Section 4.1, if Alice attempts to interfere without knowing the rearrangement order—by launching intercept–prepare–resend, intercept–measure-resend, entangle–measurement attacks—such interference will disturb the decoy states with non-zero probability, which in turn affects the protocol’s ability to detect attacks launched by potential external adversaries. This would compromise the subsequent eavesdropping detection and increase the likelihood of being discovered. Since Alice also serves as both a participant and a verifier in the eavesdropping check, any tampering she performs would undermine the protocol’s ability to ensure secure transmission and thereby increase the risk of secret information being compromised. Consequently, such attacks are self-defeating and cannot be used to disrupt the communication between Bob and Charlie.

In the second case, Alice is required to publish classical information in Step 7, which enables Bob and Charlie to infer the collapsed states of the information particles in the sequences $S_{B}$ and $S_{C}$. Based on this, each of them combines the classical data with their measurement outcomes on the encrypted sequences $S_{AB}$ and $S_{AC}$, respectively, to reconstruct Alice’s secret message $m_{A}$. If Alice dishonestly publishes incorrect measurement results, the immediate consequence is a disruption in her own communication with Bob and Charlie, rather than in the dialogue between Bob and Charlie themselves. This result contradicts her intention as a dishonest participant, which is to interfere with others’ communication while maintaining her own message intact.

It is noteworthy that Bob and Charlie, as participants, may also exhibit dishonest behavior. They could potentially launch attacks against particles traversing the quantum channel or perform dishonest operations on the received particles. However, on the one hand, due to their limited semi-quantum capabilities, any attempt to interfere with particles in the quantum channel—as analyzed in Section 4.1 regarding external attacks—would not afford them any advantage over a fully quantum-equipped Eve in evading detection. On the other hand, taking Bob as an example, he might perform Z-basis measurements on the information particles from sequence $S_{C}^{\prime }$ received in Step 5 and subsequently prepare counterfeit states opposite to his measurement outcomes instead of forwarding the original particles. Nevertheless, in Step 7, Alice measures the sequence $S_{C}^{\prime }$ to obtain $R_{C}^{\prime }$ and publicly announces the value of $H(R_{C}^{\prime })$. Since Charlie also has knowledge of $R_{C}^{\prime }$, she can verify whether the particles in $S_{C}^{\prime }$ were altered by Bob’s operations by computing and comparing the resulting values. Any inconsistency would reveal Bob’s dishonest activity. Similarly, if Charlie were to engage in analogous particle counterfeiting, her dishonest behavior could be detected by Alice and Bob in Step 7.

In summary, the protocol demonstrates strong robustness and security against internal attacks launched by dishonest participants.

### 4.3. Information Leakage

In a quantum dialogue protocol, if an external eavesdropper Eve can deduce part or all of the secret information solely based on the classical information publicly announced by legitimate participants—without launching any active attack—then the protocol is considered to suffer from a potential information leakage risk. In our proposed improved protocol, such a risk may only theoretically arise in Step 6, where Alice discloses the value of $R_{A}$ along with the corresponding initial GHZ states for each particle in the sequences $S_{B}$ and $S_{C}$. This information allows Eve to infer the collapsed states of all initial GHZ states under Z-basis measurement, denoted as $S_{A}$, $S_{B}$ and $S_{C}$. However, before being sent to Bob and Charlie, the $S_{B}$ and $S_{C}$ sequences are encrypted using Alice’s secret message $m_{a}$, resulting in the ciphertext sequences $S_{AB}$ and $S_{AC}$, respectively. Without performing any active attack, Eve has no access to the states of these sequences. Therefore, the only way she can attempt to retrieve Alice’s secret is through random guessing. For each bit of Alice’s message, Eve has a probability of 1/2 to guess correctly. According to information theory, this corresponds to an entropy of $-\sum_{i=1}^{n}P_{i}\log_{2}P_{i}=-\left(2\times \frac{1}{2}\times \left(\log_{2}\frac{1}{2}\right)\right)=1$ bit per secret bit, indicating that the classical information published by Alice does not reveal her secret.

Furthermore, Bob’s and Charlie’s messages are also encrypted within the $S_{AB}$ and $S_{AC}$ sequences, which are protected by a second round of eavesdropping detection. Without knowledge of the pre-encryption and post-encryption states, Eve can also only rely on random guessing to infer Bob’s and Charlie’s secrets. The guessing probability and information entropy for each bit remain the same, i.e., 1 bit, indicating that the classical information published by Alice does not reveal Bob and Charlie’s secrets.

In summary, there will be no information leakage problem in the improved protocol.

## 5. Efficiency Analysis

Qubit efficiency is one of the key indicators for evaluating the practicality of a quantum communication protocol. It can be formally calculated using the efficiency formula proposed by Cabello [35]: $\eta =\frac{b_{s}}{q_{s}+q_{t}}$ where $b_{s}$ denotes the total number of secret bits successfully transmitted, and $q_{s}$ and $q_{t}$ represent the total number of qubits and classical bits consumed for transmitting these secret messages, respectively. Note that decoy photons and classical bits used solely for eavesdropping detection are excluded from this calculation. Table 1 summarizes a comparative analysis of our proposed protocol with several existing three-party QD protocols.

In protocol [15], all three participants possess full quantum capabilities and follow a symmetric communication model, where each participant is able to both send and receive *n* bits of secret information to and from the other two participants. This results in a total of 6*n* secret bits exchanged. Alice prepares a sequence of *n* continuous-variable GHZ states to carry the secret messages, consuming 3*n* qubits. For classical communication, each participant announces their measurement results twice for a sequence of *n* particles, yielding a total of 3*n* classical bits. Thus, the overall quantum bit efficiency of protocol [15] is $\eta =\frac{6n}{3n+6n}\approx 0.667$.

In protocol [16], the system includes a semi-honest third party (TP) and three fully quantum-capable participants, who engage in symmetric communication. Each participant is able to send and receive *n* bits of secret information to and from the other two, resulting in 6*n* secret bits exchanged. TP prepares a sequence of *n* four-dimensional four-particle entangled GHZ states to carry the secret messages, resulting in a consumption of $q_{s}=\log_{2}4\times 4\times n=8n$ qubits. No classical bits are used for transmitting the secret messages. Therefore, the total quantum bit efficiency of protocol [16] is $\eta =\frac{6n}{8n}=0.75$.

In protocol [28], the communication scenario involves one fully quantum participant, Charlie, and two semi-quantum participants, Alice and Bob. The communication pattern is asymmetric, as it enables bidirectional message exchange only between Charlie and Alice (or Bob), while direct communication between Alice and Bob is not supported. In this protocol, both Alice and Bob transmit *n* bits of secret information to Charlie, and Charlie likewise sends *n* bits to each of them, yielding a total of 4*n* secret bits transmitted. To enable this, Charlie prepares 2*n* four-particle cluster states, corresponding to 8*n* qubits used. Additionally, Charlie discloses 2*n* classical bits to publish his encoding sequences. As a result, the overall qubit efficiency of this protocol is $\eta =\frac{4n}{8n+2n}=0.2$.

In contrast, the original ZZL-SQD protocol adopts a symmetric communication model, where each participant is able to both send and receive *n* bits of secret information to and from the other two participants. This yields a total of 6n secret bits exchanged. Alice prepares a sequence of *n* GHZ states to carry the secret messages, resulting in 3*n* qubits. For classical communication, Alice publishes 3*n* bits to reveal the initial GHZ states, *n* bits for Bob’s and Charlie’s secret message sequence, and another *n* bits for her own message sequence, summing up to 5*n* bits. Thus, the total efficiency of the ZZL-SQD protocol is $\eta =\frac{6n}{3n+5n}=0.75$.

Our improved protocol also adopts a symmetric communication structure, maintaining full two-way information exchange between all three participants. Each of them receives *n* bits of secret messages from the other two, again yielding a total of 6*n* bits. Alice prepares *n* GHZ states for message transmission, requiring 3*n* qubits. The classical communication involves the publication of 3*n* bits to indicate the initial GHZ states and *n* bits for the measurement results of particle sequence $S_{A}$, giving a total of 4*n* bits. Consequently, the qubit efficiency of the improved protocol reaches $\eta =\frac{6n}{3n+4n}\approx 0.857$.

These results demonstrate that, in addition to providing enhanced resistance against insider attacks, our improved protocol also achieves a significant increase in quantum communication efficiency compared to both the original ZZL-SQD protocol and other protocol, thereby offering greater practicality and scalability in real-world quantum communication scenarios.

## 6. Conclusions

In summary, this work first provides a brief overview of the original ZZL-SQD protocol and presents a rigorous analysis demonstrating its vulnerability to attacks from dishonest participants. To address this issue, we propose an effective improvement scheme. The improved ZZL-SQD protocol ensures symmetric communication among participants without requiring any enhancement of their quantum capabilities. From a security standpoint, the protocol addresses the original scheme’s inability to resist dishonest participant attack, and further demonstrates robustness against various external and internal attack scenarios, while effectively preventing information leakage. In addition, the improved protocol achieves a notable enhancement in quantum communication efficiency, making it significantly more practical and feasible for real-world applications compared to the original protocol.

## Figures and Tables

**Table 1:** The comparison with some existing three-party QD protocols.

	[15]	[16]	[28]	ZZL-SQD Protocol	This Work
Protocol Type	Fully Quantum	Fully Quantum	Semi-Quantum	Semi-Quantum	Semi-Quantum
Communication Pattern	Symmetric	Asymmetric	Asymmetric	Symmetric	Symmetric
Quantum Resources	Continuous variable GHZ states	4-dimensional 4-particle entangled GHZ states	Four-particle cluster states	GHZ states	GHZ states
Analysis dishonest participant attack	No	Yes	Yes	No	Yes
*b_s_*	6*n*	6*n*	4*n*	6*n*	6*n*
*q_t_*	3*n*	8*n*	8*n*	3*n*	3*n*
*q_s_*	9*n*	0	2*n*	5*n*	4*n*
*η*	66.7%	75%	20%	75%	85.7%

## Data Availability

The original contributions presented in this study are included in the article. Further inquiries can be directed to the corresponding author.
